# Lockjaw the Result of Vaccination

**Published:** 1899-10

**Authors:** 


### LOCKJAW THE RESULT OF VACCINATION.

Carl, the eight-year-old son of Carl Pfeffer, Sr., a shoemaker, of Millvale, is dying (Sept. 9th) of lock-jaw, caused by vaccination. The case is one of the most remarkable in the history of medical practice in Allegheny County and has excited the wonder of the physicians who have attended the boy.

The Pfeffer boy was vaccinated three weeks ago Thursday by Dr. A. K. Lyon, a reputable physician of Millvale, who sailed for Berlin, Germany, yesterday, to take a year's post-graduate course in medicine. Thursday morning the boy was taken ill. The first symptom was the hardening of the muscles of the jaw and the back of the neck. As the trouble did not disappear, Dr. T. M. Fife, an experienced physician of the town, was sent for. He immediately diagnosed the case as genuine tetanus, and, failing to find evidence of any other cause, decided it was due to the effects of vaccination, performed three weeks before.

To satisfy himself on this point, however, Dr. Fife consulted with Dr. J. M. McCready, of Allegheny, who called and saw the boy. Dr. McCready also pronounced it lockjaw and gave it as his opinion that it was caused by the scratching of the boy's arm by the doctor's lance. There is quite a deep impression on the left arm where it was vaccinated, but Dr. Fife says it is not as large as other marks he has seen.

The boy has grown worse rapidly since Thursday morning, and yesterday afternoon was not expected to live longer than to-day. His jaws locked Thursday and he passed from one convulsion to another every five minutes. The doctor succeeded, with great efforts, in forcing some medicine down his throat, and the spasms came with less frequency.

Yesterday he lay in a stupor, had not been able to receive any nourishment and was suffering intensely. His fever was 106, and the convulsion occurred about every ten minutes. In the afternoon a trained nurse from Allegheny was secured to help the worn-out father and mother, who had been watching and nursing their son day and night. The boy's jaws became more tightly locked yesterday and his temperature increased several degrees. All the attendants and physicians could do was to keep ice packed around his head and face to reduce the high fever as much as possible and inject medicine and milk through the teeth by means of a straw.

The Pfeffers are a highly respected German family and live in a neat double-story frame house on Stanton Avenue extension. The stricken lad is a bright-eyed, light-haired little chap and was the pet of the family.

The case has caused considerable alarm in Millvale, where over a thousand children have been compelled to undergo vaccination before being admitted to the public schools.

Dr. Fife told a *Dispatch* reporter that there were quite a number of people in Millvale who have been opposed to vaccination and he thought the Pfeffer case would tend to confirm their opinions and those of other anti-vaccinationists, that the operation is both dangerous and useless. He said he had hesitated to decide that tetanus had resulted from vaccination, but he saw no other alternative, although it was the first that he had ever known or read of.

He said no blame could be attached to Dr. Lyon, as he was a physician of high standing in his profession, and, like all other doctors of Millvale, had performed his vaccinations with virus obtained from the Pittsburg Bureau of Health.

Dr. E. G. Matson, of the city bacteriological department, was seen last evening in reference to the Pfeffer case. He is of the opinion that some other cause than vaccination produced lockjaw. He thinks that a further investigation of the case will show that his view is correct. "Lockjaw," said he, "is produced by a tetanus bacillus, a germ which poisons the blood. It is quite common, and it is surprising that more deaths are not caused by it. It exists in the earth and is very dangerous when introduced into the system. If the boy had no other wounds on him, it is possible that the tetanus germ may have gotten into the vaccination sore, but not through the vaccine virus. We buy our quills from a New England company, and this is the first case that has come under my notice causing any trouble. I do not even know that the vaccine used in this case was purchased from us. Vaccine virus is sold at nearly all the drug stores and the physician may have gotten it somewhere else.

"There have been more cases of lockjaw this year in Pittsburg than ever before. The reports show that seven people have died as a result of the disease. This is not because the tetanus germs are more numerous, but because the conditions are more favorable. In every instance the victim sustained some serious wound through which the germ was introduced. The Pfeffer boy may have suffered an abrasion of the skin or a puncture which healed hurriedly with the tetanus germ inside. In time this would develop and pass into the blood, causing death from lockjaw. Even if it were possible that the vaccination in this case caused lockjaw it is not to be compared to an epidemic of small-pox which the operation will avert. The public need not be alarmed, and I believe, as I first stated, that some other cause than vaccination caused the lockjaw in this instance."—*Pittsburg Despatch*, September 9th, 1899.

